# Metallographic Testing of 19th Century Steel in an Operating Water Tower

**DOI:** 10.3390/ma14092204

**Published:** 2021-04-25

**Authors:** Eugeniusz Hotała, Rajmund Ignatowicz, Maciej B. Lachowicz

**Affiliations:** 1 Department of Building Structures, Faculty of Civil Engineering, Wroclaw University of Science and Technology, Na Grobli 15, 50-421 Wroclaw, Poland; eugeniusz.hotala@pwr.edu.pl; 2 Department of Metal Forming, Welding Technology and Metrology, Faculty of Mechanical Engineering, Wroclaw University of Science and Technology, Lukasiewicza 7-9, 50-371 Wroclaw, Poland; maciej.lachowicz@pwr.edu.pl; 3 Machinefish Materials & Technologies Sp. z o.o., Dunska 13, 54-027 Wroclaw, Poland

**Keywords:** weldable iron, puddled steel, corrosion, microstructure, chemical composition, hardness, steel water tank, water supply systems

## Abstract

The world’s first steel structures were built towards the end of the 19th century. Some of them are still in use today, whereas others are maintained as precious technical heritage. In both cases, there may be a need to assess their technical condition and carry out repairs and reinforcements, which requires an understanding of the properties of the steel used. The few studies that have been undertaken of such steel structures indicate that the properties depend on the history of use. This paper presents the results of metallographic tests of a steel tank in a water tower built in 1884 in Lower Silesia. The chemical composition was consistent with that of the puddled steel used in the 19th century. The carbon content showed significant segregation and ranged from 0.011% to 0.072% mass. As a consequence, a typical microstructure for low-carbon steels (ferritic) was observed, changing locally to ferritic-pearlitic. The tested steel contained a very high content of phosphorus and silicon. The microstructure with numerous slags favoured the formation of surface delamination caused by the corrosion processes. The degree of corrosion of the steel of the tank was also assessed, as well as the type of corrosion inside the tank. Corrosion was favoured by the oxygen concentration cell. The results of the research will be used to assess the potential for continuing tank use and the reinforcements that have been planned. The results presented will add to the somewhat limited research results available for steel dating back to the 19th century, which is still present in many building structures. Such a database is especially needed by those designing technical measures aimed at maintaining these historical structures in good technical condition.

## 1. Introduction

The long-term use of steel structures leads to significant microstructural changes. These are an important challenge in buildings which have been in use for long periods. For this reason, there is great interest in the literature when it comes to the material testing of structures made from old low-carbon steel [1,2,3,4,5,6,7,8,9,10]. Studies have indicated that the degradation of microstructure due to corrosion processes leads to a decrease in steel ductility, as well as to changes in the character and rate at which fatigue cracking develops [1,2]. A practical indicator describing the degradation processes taking place in steel is the coefficient σ_0.2_/σ_u_ [3]. The degradation of steel leads to an increase in the deformation reinforcement co-efficient [4]. The stress–strain graph also indicates the appearance of a plateau (shelf) of plasticity [4]. Degradation changes may become intensified due to the influence of the corrosive environment, especially under hydrogen influence conditions [5,6]. The degradation of the microstructure favours increasing sensitivity to hydrogen embrittlement [4].

**Figure 1:**
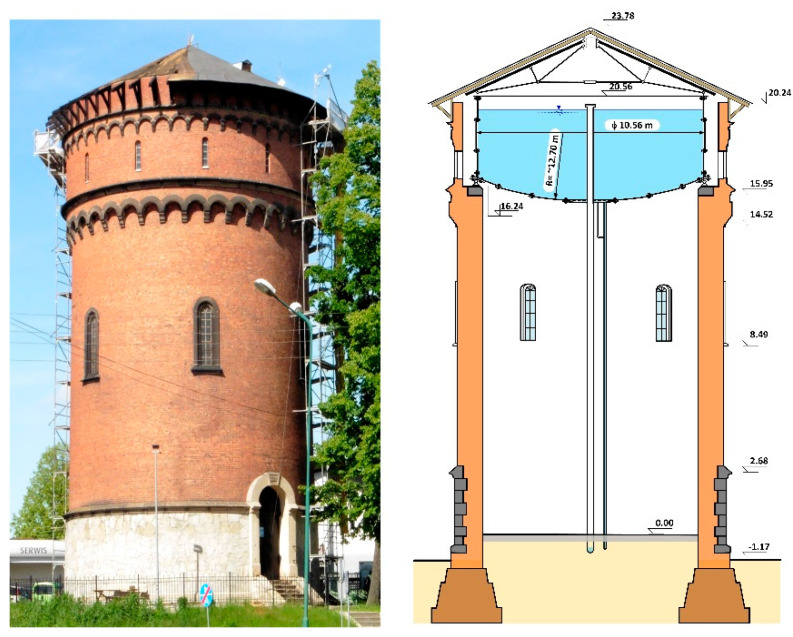
View and section of the pressure tower.

**Figure 2:**
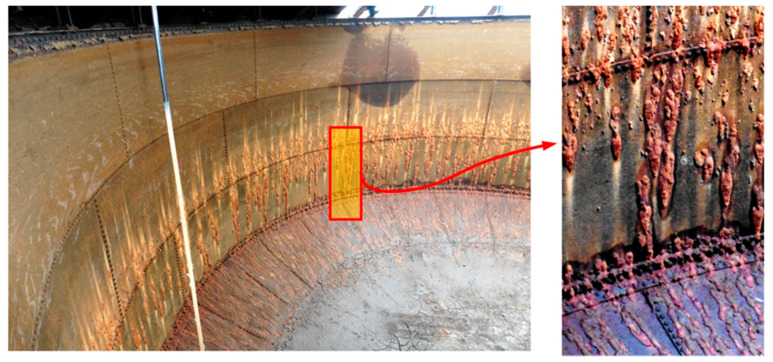
View of a fragment of the interior of the steel tank in the tower.

In the second half of the 19th century, many water towers were built in Lower Silesia. There were made then with weldable steel (puddled steel), which was also called puddled iron [1,2]. They were some of the first iron shell structures in the world. The production of metal sheets made of puddled steel and cast steel did not start until the second half of the 19th century. At that time the first coated structures were introduced in cylindrical water tanks. In 1884, a steel water tank with a total capacity of 400 m^3^ was built as part of a pressure tower in Lower Silesia (Figure 1 and Figure 2) and has been used for this purpose without interruptions until today. 

The technical condition of the steel jacket and bottom of the steel tank were tested after 135 years of uninterrupted use to determine the degree of corrosion wear and the potential for continuing use. The steel’s microstructure was tested. Spectral analyses of the chemical composition were carried out, as well as other material tests. The results of the material testing provided a basis for assessing the corrosion processes taking place in the steel sheets of the tank and their strength parameters, and so, for decisions as to allowing the continued use of the tank in coming years. The test results of steel from the tank structure are presented in this paper. The test results concerning the properties of 19th century steel may be helpful in assessments of durability and suitability for use on other historical structures made from old structural steels. The use of real material data may be of practical importance in the experimental modelling and forecasting of the residual lifetime of structures made from such steel [7,8,9]. In addition to their historical value, such historical structures may also provide good performance parameters. In fact, the residual life of historical steel structures is often much longer than that of similar structures built today. The oldest steel silos in the world, which are presented in this paper, are a case in point [11].

## 2. General Description of the Tank Structure and Its Technical Condition

**Figure 3:**
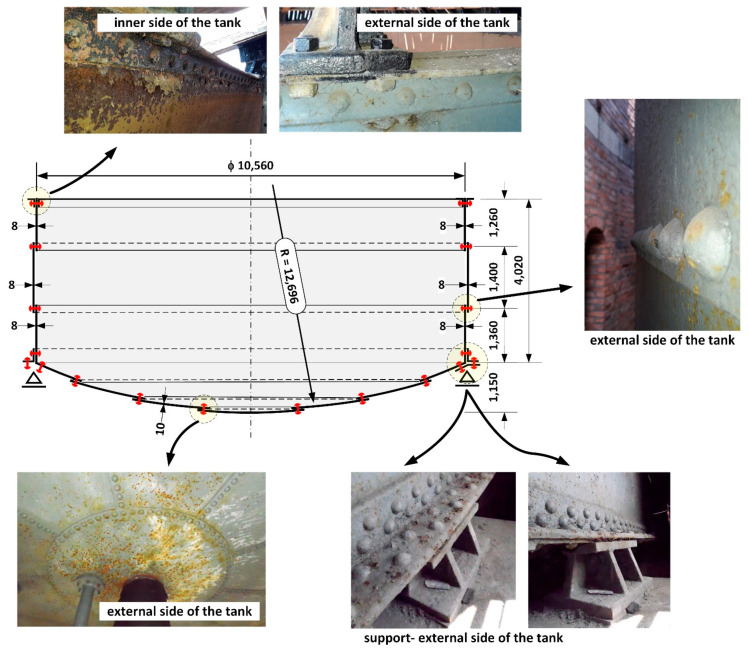
Geometry of the steel water tank. The design steel sheet thickness is indicated.

Testing concerned a steel cylindrical tank of diameter D = 10,560 mm and shell height H = 4020 mm and a bottom spherical-shaped cap of radius r = 12,696 mm (Figure 3). The shell of the tank was made of metal sheets t = 8–10 mm thick and the bottom comprised metal sheets of t = 10 mm thick. The sheets were joined together with rivets with pin diameter of d = 22 mm.

The tank was filled with water every day during the night. During the day the tank supplied water to the water supply system, maintaining the almost constant pressure required. Over the past 30 years of operation, the tank was almost completely emptied and refilled every day. This means that over 135 years there may have been more than 40,000 alternating water load cycles, although it is impossible to recreate a more precise operational history.

**Figure 4:**
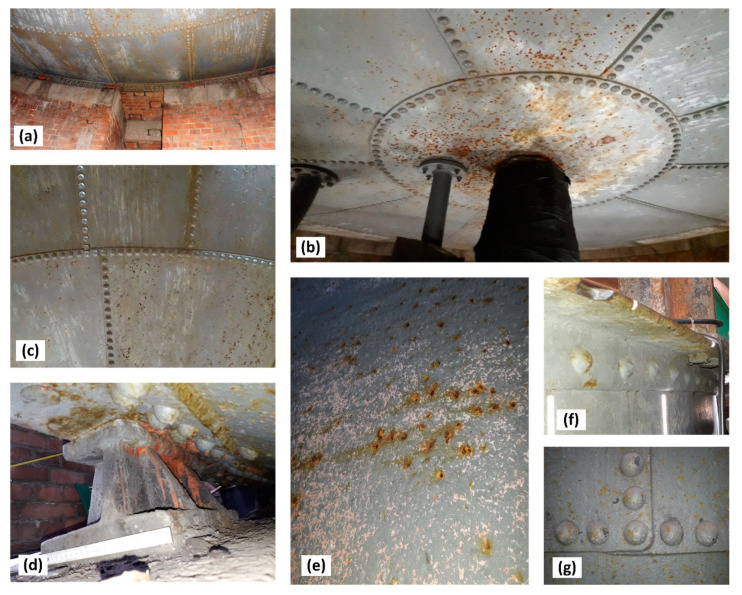
Condition of the outside surface of the sheet steel of the tank: (**a**–**c**) the tank bottom, (**d**) tank support, (**e**–**g**) tank wall.

During renovation of the pressure tower roof (Figure 1), the steel tank was emptied (Figure 2) and the corrosion wear of the metal sheets was tested from the inside, as these were clearly visible. From the external side of the tank, the corrosion process was not so intense (Figure 4) given 135 years of operation.

The corrosion was found to be electrochemical in character and evenly distributed, but with local pitting corrosion up to a depth of 1.0–1.5 mm. The highest corrosion losses in the steel sheet of the cylindrical sides of the tank did not exceed 2.5 mm, whereas in the steel sheets of the spherical bottom they did not exceed 1.0 mm, taking into account also localised corrosion pits. The bottom of the tank was almost always covered with a layer of water, as the tank was very rarely emptied completely, hence the corrosion rate of the bottom steel was almost always low. The steel sheets of the tank shell were exposed to atmospheric corrosion on a daily basis. This was because when the water level in the tank fell during the day, so the shell remained covered with a thin layer of water, which together with the substances dissolved in it formed an electrolyte. Every night the tank was filled completely with water.

## 3. Research Methodology

The following test methods were applied to study the tank steel:

(1) The chemical composition was determined by means of the spectral method using the LECO GDS-500A (Leco Corporation, St. Joseph, MI, USA) analyser with glow discharge (GDOES).
(2) The microscopic tests were performed using the Leica M205 C stereoscopic microscope (Leica Microsystems, Wetzlar, Hesse, Germany), Leica DM6000 M metallographic microscope (Leica Microsystems, Wetzlar, Hesse, Germany), as well as the Phenom World ProX scanning electron microscope (Thermo Fisher Scientific, Waltham, MA, USA), equipped with the EDS detector on the conventionally prepared metallographic specimens. The tests were carried out on samples in a non-etched and etched with 2% Nital solution state.
(3) The HV1 hardness measurements were performed using the Vickers method according to the PN-EN ISO 6507-1: 2007 standard with the application of a 1 kg load. The test was carried out with the use of a LECO LM-248AT (Leco Corporation, St. Joseph, MI, USA) hardness tester.

## 4. Results of Tests of the Chemical Composition of Tank Steel

Only a few publications [12,13,14] have been concerned with the mechanical properties and chemical composition of structural steels dating back to the end of the 19th century. It is important to distinguish between the so-called puddled iron (German Schweisseisen) and the cast steel (German Flussstahl) produced at that time in steelworks in Austria and Germany. The chemical composition of these old steel types is shown in Table 1 (with total extended uncertainty U for the confidence level of about 95% and extension coefficient k = 2). The result is given as the mean of five determinations.

**Figure 5:**
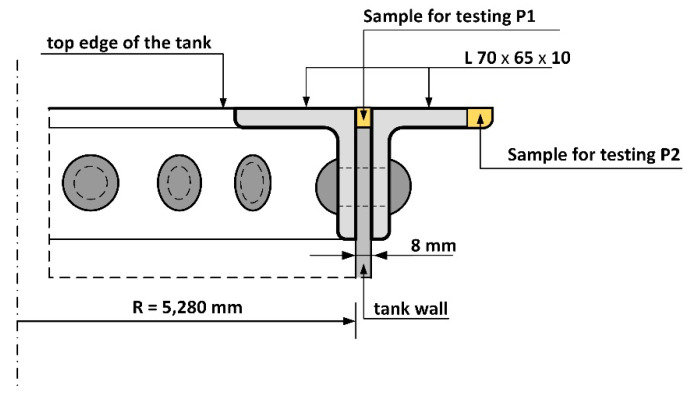
Steel sampling locations P1 and P2 on the upper edge of the tank.

A spectral analysis was carried out to assess the material type of the tank band. The tank owner allowed steel sampling only in the top edge of the tank, but it is highly likely that the material test results obtained are representative for the tank as a whole (Figure 5). Based on the analysis, it was determined that the chemical composition was consistent with that of puddled steel used in the 19th century. The test results obtained for the band material of the tank are presented in Table 1. The values presented are an average of four individual analyses. There is no currently available equivalent to this type of steel. The carbon content obtained from the four analyses ranged from 0.011% to 0.072%, which is a significant spread. However, the results obtained correlate with localised microstructure changes in the tested material, which is mostly a ferritic microstructure containing approx. 0.0218% carbon. A ferritic–perlite microstructure with a higher carbon content can be found in some areas. Segregation of some elements is a characteristic feature of puddled and cast steels, especially carbon, phosphorus and sulphur. A very high phosphorus content was found in the steel tested, exceeding by three times the amount of phosphorus in structural steels produced today. High phosphorus content in puddled steel has been observed by other researchers [1,2,7,9,10]. The authors of the paper [9] found that the high silicon content was linked to processes of steel deoxidation or desulphurization. This situation favours degenerative processes in these steels [1].

## 5. Results of Metallographic Tests of the Tank Steel

**Figure 6:**
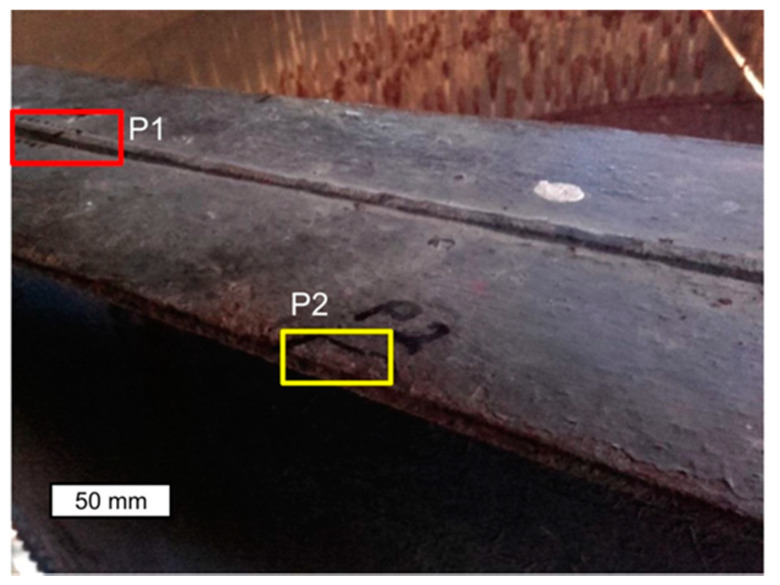
Sampling locations P1 and P2 from the tank wall for samples used for material testing. The dashed line indicates cross-sections designated for microscope observation: longitudinal cross-section—red colour; transversal cross-section—yellow colour.

**Figure 7:**
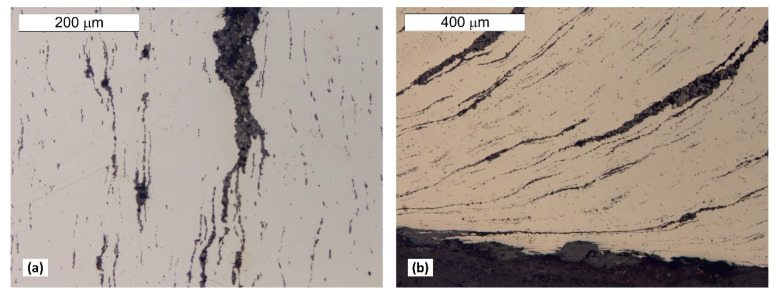
Longitudinal cross-section (**a**) and lateral (**b**) samples P1 taken from the tank wall. Numerous banded impurities and heterogeneity indicating their complex structure. Light microscope, non-etched state.

**Figure 8:**
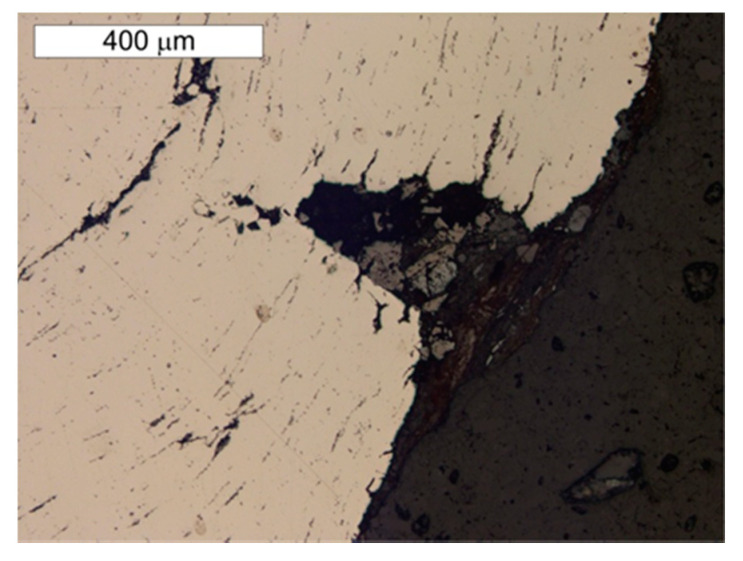
Transversal cross section of sample P1 taken from the tank wall. Numerous banded slag impurities are visible. A layer of corrosion products is visible on the surface, penetrating into the wall material interior at specific locations. Light microscope, non-etched state.

Material tests were carried out after long-term exposure. Samples for testing were taken in locations indicated in Figure 6. Microscope analysis indicated the presence of significant amounts of slag impurities in the material microstructure of the angle section tested (different in size and morphology), which is a characteristic feature of old weldable steels (Figure 7 and Figure 8). The chemical composition of the steel is conducive to the formation of precipitations composed mainly of phosphorus and sulphur. However, oxide inclusions are also common in these materials [7]. Slag inclusions are distributed in bands, longitudinally to the direction of metal forming. The transversal cross-section provided a visible orientation of the microstructure in the direction of plastic deformation, resulting most probably from the shaping of the tank shell edges. A corrosion products layer is visible on the surface. They locally penetrate deep into the material of the tank wall (Figure 8).

The microstructure present in the material will favour the formation of surface delamination caused by the corrosion processes. Such delamination was also observed by Panasyuk et al. [5] in the microscope image.

**Figure 9:**
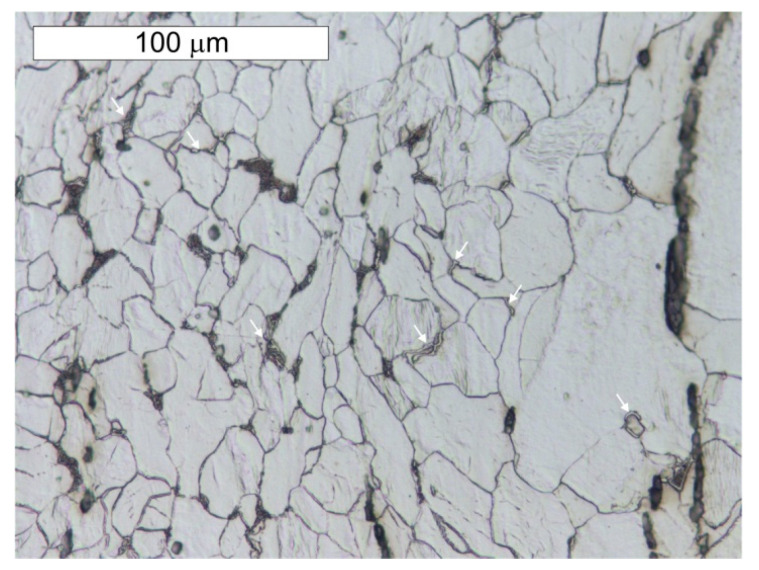
Longitudinal section of sample P1 taken from the tank wall. Tertiary cementite and degenerated perlite formed as a result of material degradation are marked with arrows. Light microscope, etched state.

**Figure 10:**
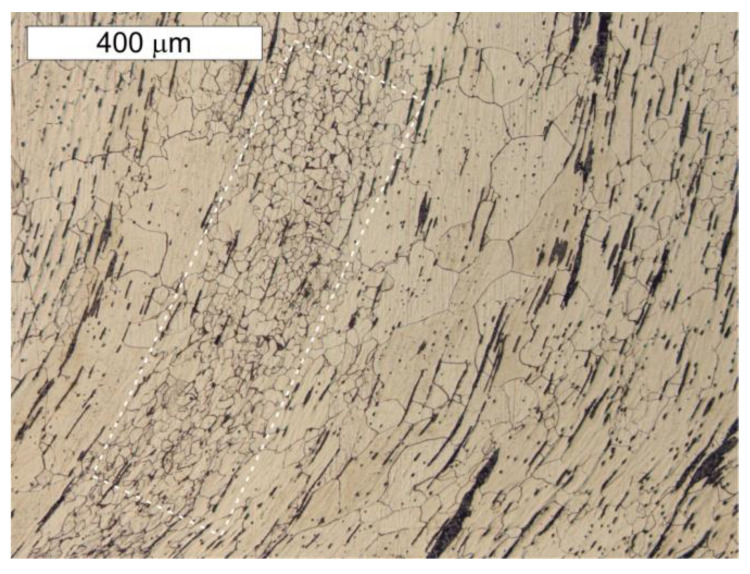
Cross section of sample P1 taken from the tank wall. Numerous bands of slag impurities are visible. The ferritic microstructure predominates with heterogeneous grain with localized areas of ferritic–perlite microstructure (frame). Light microscope, etched state.

**Figure 11:**
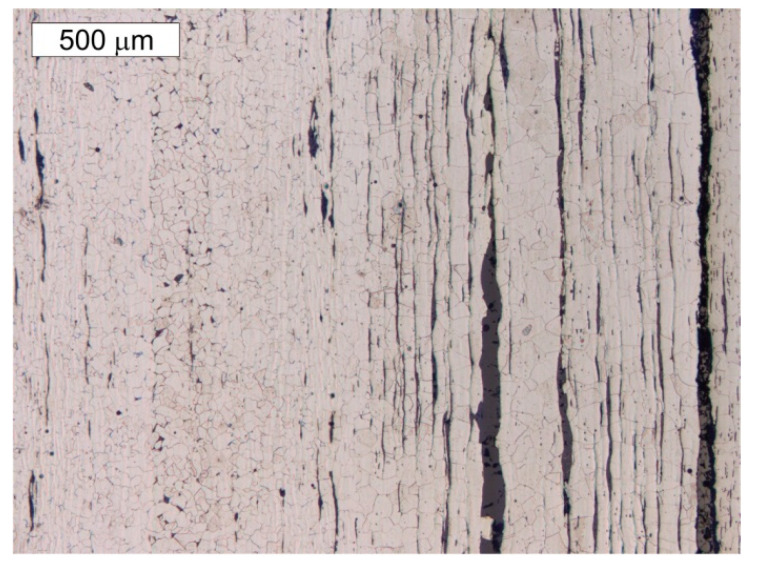
Longitudinal cross-section of the P2 sample taken from the angled section of the tank band. Ferritic microstructure with few perlite grains. Numerous bands of slag impurities are visible. Light microscope, etched state.

**Figure 12:**
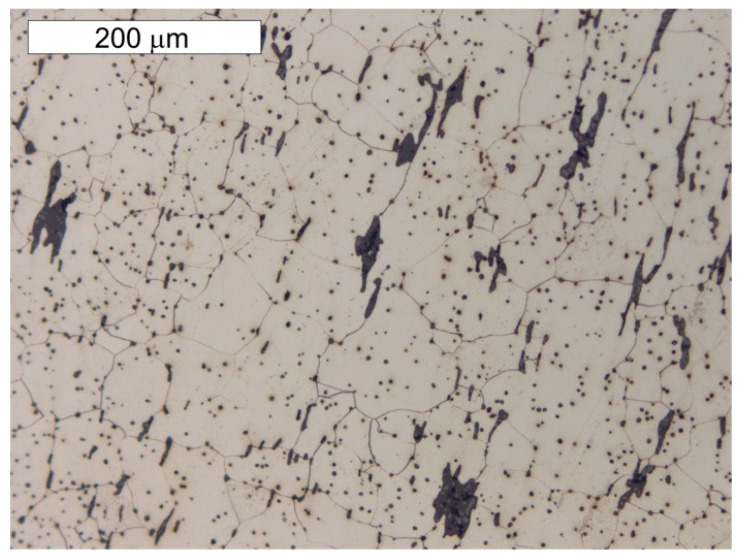
Lateral cross-section of sample P2 taken from the angle section of the tank band. High degree of slag impurities is visible in the area of the ferritic microstructure. Light microscope, etched state.

**Figure 13:**
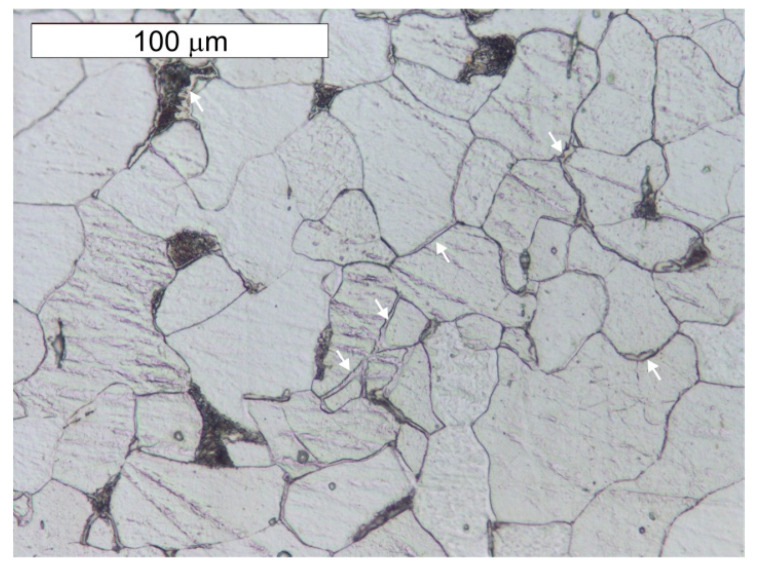
Longitudinal cross-section of the P2 sample taken from the angle section of the tank band. Tertiary cementite and degenerated perlite formed as a result of material degradation are marked with arrows. Light microscope, etched state.

A microscope analysis carried out in the etched state indicated a mostly ferritic microstructure, but zones of ferritic–perlite microstructure were also observed, as presented in Figure 9 and Figure 10. The ferritic–perlite microstructure is typical for low carbon puddled steel [2,7,9,10]. The simultaneous occurrence of ferritic and ferritic–perlitic zones indicates a different carbon content in the steel, which is typical for puddled steels. Structural elements made of puddled steels display a significant tendency for microstructure degradation [2,10]. In microscopic examinations, numerous precipitations of tertiary cementite were found at the grain boundaries of the ferrite, the presence of which indicates a considerably advanced steel ageing processes [1,3]. Degenerate perlite was also observed locally. This process is related to the decomposition of perlite into ferrite and carbides, which is also associated with degenerative processes taking place in these steels [3]. In the case of unalloyed steel, it should be expected that the perlite decomposed into ferrite and cementite. The ferrite grains showed considerable variation in size. Such microstructural heterogeneity favours the initiation of fatigue cracks [7]. The microstructure of the steel sample taken from the angled section of the band (sample P2) was similar to the microstructure of the steel taken from the tank shell (sample P1)—Figure 11 and Figure 12. The transversal cross-section of sample P2, however, indicated no grain elongation in the direction of plastic deformation, which had been observed in the same cross-section of sample P1 (Figure 12). It was most probably caused by the plastic bending of the upper edge of the tank shell. The strain hardening caused by this resulted in the hardness differentiation of the water tank shell material and the angled section of the tank band, which showed an average hardness of 195.1 ± 14.8 HV1 and 132.9 ± 4.4 HV1, respectively. Similarly to in the case of the P1 sample, numerous precipitations of tertiary cementite were observed at the grain boundaries of ferrite and degenerated perlite, which are associated with the long-term degenerative processes occurring in these types of steel (Figure 13).

## 6. Assessment of Corrosion Intensity of Shell and Tank Bottom Steel

**Figure 14:**
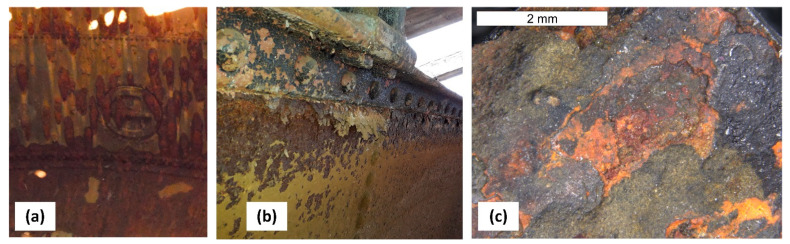
Corrosion products taken from the tank wall: (**a**) tank walls at the inspection deck, (**b**) top edge of tank wall, (**c**) corrosion area in the stereoscopic image.

**Figure 15:**
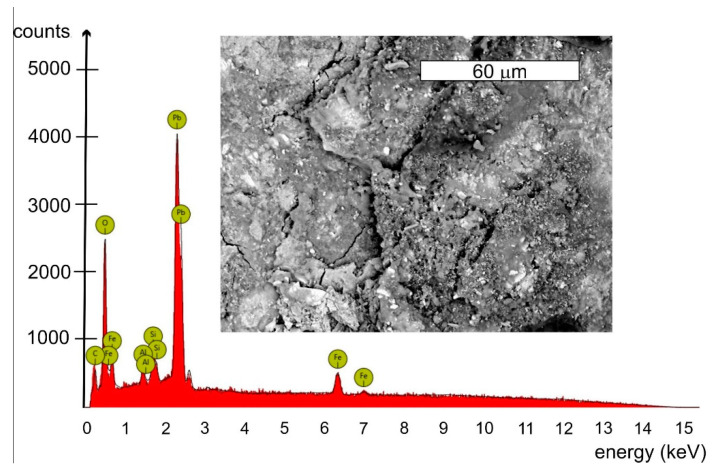
Example of EDS spectrum obtained for the area shown in the microscope image.

Features of the intensive corrosion were visible on the inner surface of the cylindrical shell steel of the empty tank. The corrosion products were distributed evenly over the entire surface of the steel sheet and localized in the form of clear thickening of so-called “corrosive braids” (Figure 2). The reaction of corrosion products was pH = 6.5. The microanalysis of the chemical composition of corrosion products inside of the tank was determined using the EDS method. A general view of the corrosion products was presented in Figure 14. The EDS microanalysis was performed in several randomly selected places. Similar results were obtained. An example spectrum of characteristic X-rays is presented in Figure 15. The results of the microanalysis of the chemical composition performed in this area were shown in Table 2. The tests found that the corrosion products were rich in lead, with mass concentrations ranging from approx. 20% up to even 50%. Old water tanks were sealed with thin overlapping lead sheets, and many pipes in water supply systems were made of lead until the mid-20th century. Leonetti et al. [16] showed that the presence of the red-lead paint ensured high air-tightness of two plate-to-plate contacting surfaces. It also contributes to the reduction of the friction coefficient between the plates, which affects the fatigue strength of the joint [17]. It cannot be ruled out that the tank has not been protected with anticorrosive coatings over the years of operation. The steel sheets of the tank could be covered with an anticorrosive paint containing lead oxides. This may explain the occurrence of lead in the corrosion products inside the tank. The presence of lead in a dense layer of corrosion products, large tank capacity, daily water exchange, as well as a stagnant character are all factors that limit the possibility of lead ions passing into the water. This was confirmed by the fact that the tank user did not detect the presence of lead in the drinking water.

Microbiologically Influenced Corrosion (MIC) may occur in water tanks, the course and effects of which are described in various papers, among others [18,19]. Aside from a significant amount of oxygen associated with the formation of iron oxides and high lead content, the EDS microanalyses of the corrosion products inside the tank also detected small amounts (1–3%) of zinc (Zn), calcium (Ca), silicon (Si) and aluminium (Al). A similar origin related to anticorrosion protection as in the case of lead can be attributed to zinc and aluminium observed in the EDS spectrum. No significant sulphur content was found in these products. This suggests that the corrosion is unlikely to be caused by biofilm induced by sulphate-reducing bacteria (SRB) (Figure 14).

The corrosion is clearly electrochemical atmospheric and even in character with locally occurring pits. Atmospheric corrosion occurs when the air humidity is above 70%, resulting in a thin water layer forming on the surface of the steel tank sheets, which together with the substances dissolved in it form an electrolyte. Data from the literature indicate that advanced uniform corrosion may contribute to stability loss of steel tank shells [20].

In the tested tank, a water layer formed on the inside as a result of the daily lowering of the water surface in the tank. The wall of the tank shell above the water line was thus wet for part of the time. An important role is played in this situation by differential aeration cell to individual fragments of the metal surface. The zones submerged in water are anode areas, whereas those above the water line are cathodic. This situation forms oxygen aeration cell, which intensifies localised corrosion processes. The tank bottom has been exposed quite rarely with the result that corrosion processes impacting the inner part of the spherical bottom are much less advanced than those the sheets of the shell (Table 1).

Features of atmospheric corrosion of the tank bottom and tank shell were found on the external side of the riveted tank (Figure 4). Localised flaking of the anticorrosive paint coating and localised corrosion pits are visible, but their intensity is not high.

**Figure 16:**
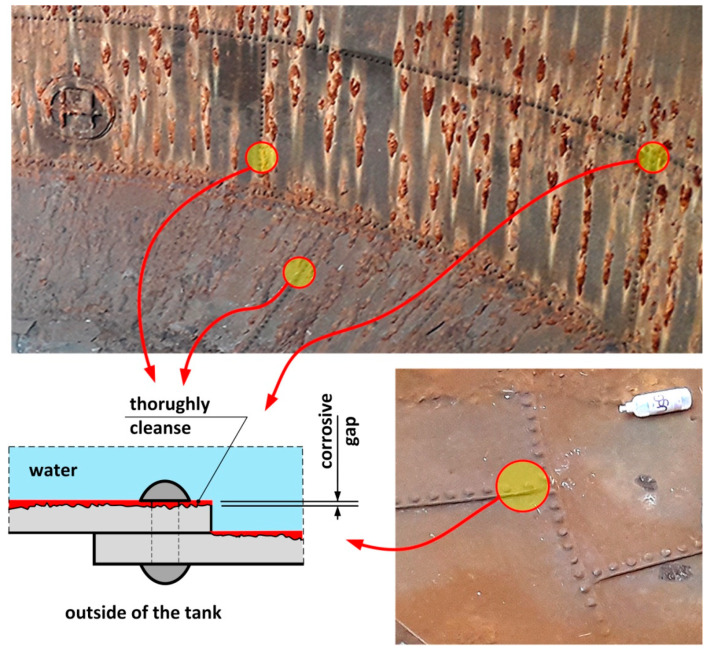
Possible case of corrosion of sheets beneath the rivets.

Measurements of the thickness of the metal sheets of the tank shell and bottom were carried out using a calibrated ultrasonic thickness gauge with a measurement accuracy of 0.1 mm. Measuring the thickness of tank sheets was difficult due to a layered structure of sheets with large slag impurities between layers. As a result, a detailed visual inspection was carried out of the corrosion state of sheets around rivets (Figure 4). The hot riveting process was used extensively up to about 1970 for joining plates in built-up cross-sections [16]. At the stage of manufacturing the rivet (during cooling after forging), the rivet shank was shortened, which led to an increase in the yield stress and tensile strength of the steel [16,17]. The joined plates were thus pressed against each other by the rivet heads, which caused an internal axial force to be created, referred to as the “clamping force”. Literature data indicate that its value ranged on average from 60 to 100 MPa [16]. The clamping force induced by the hot riveting technique is higher than that obtained with prestressed bolts [21]. It also prevents the occurrence of their self-loosening. No gaps were found between the rivet heads and the tank shell sheets, as presented in Figure 16. In consequence, it was easier to reject the very small measures of sheet thickness, as these were determined as resulting from the layered structure. With respect to corrosion of the sheets around the rivet heads, the sealing could have loosened at contact points resulting in water leaks from the tank. Such a situation did not occur in the tested tank.

Localised corrosion pits of about 1–1.5 mm in depth were found. Under the thick “braids” of corrosive products on the sheets of the shell, the corrosive pits were approx. 1 mm larger than in other locations. The formation of braids was related to the nature of the microstructure, which favoured the formation of corrosive pits. The presence of non-metallic inclusions in the microstructure favours the formation of delamination, as indicated earlier. This led to the retention of the corrosive solution in these locations, which supported the electrochemical process.

The maximum measured value of local corrosion losses from the tank shell sheets amounted to 3 mm almost across its entire height. The highest part of the tank shell was originally 8 mm thick, but the current minimum thickness of 5 mm is still a value that ensures sufficient tank loading capacity. The lower parts of the shell had an original thickness of 10 mm and the maximum localised corrosion losses do not threaten safe tank operation with respect to loading capacity.

The tank bottom sheets had an original thickness of 10 mm, but showed only slight corrosion losses, not exceeding 1 mm. This is due to the fact that there was almost always a layer of water covering the bottom, making atmospheric corrosion impossible.

The estimated linear function of the corrosion progress V_p_, calculated as the loss of sheet thickness over 135 years of operation, amounted to V_p_ = 3.0/135 = 0.022 mm/year (V_p_ = l/t, where: l-corrosion loss on the sample [mm], t-time [years]). This means that in terms of the commonly used classification of metal resistance to corrosion, the sheets are of uniform corrosion-resistant steel. This does not mean, however, that this steel is completely resistant to pitting corrosion. It should also be noted that the corrosion rate will not be constant under these conditions. The degradation changes taking place in the microstructure of steel affect the rate of its corrosion, making it more susceptible in a corrosive environment [4,5,6].

## 7. Conclusions

A review of the structural safety of historic steel structures requires an understanding of their construction, the methods used to fabricate the structural elements, and the properties of the materials used. Tests carried out on steel in a water tank which has been operating for 135 years lead to the following conclusions:

1. The chemical composition of tank steel indicates that a puddled steel was used to build the pressure tower tank. Moreover, the ferritic–perlite microstructure with considerable heterogeneity and numerous non-metallic inclusions and slag is typical for this group of materials. In the microstructure, there are also numerous precipitations of tertiary cementite at the ferrite grain boundaries, the presence of which indicate advanced steel ageing. Localised degenerate perlite was also observed. This is related to the decomposition of perlite into ferrite and carbides, which is also associated with degeneration processes taking place in these types of steels.
2. The water tank should be classified as unfit for welding on account of the high phosphorus content, which is limited to 0.035 ÷ 0.05% in weldable low alloy steels, numerous slag non-metallic inclusions and the presence of tertiary cementite. This is a significant problem when riveted parts of a steel tank will need to be replaced. Riveted joints ensured a secure fit of the plates to be joined, despite their corrosion. Searching for the possibility of joining steel plates in the case of their renovation with the use of prestressed bolts requires their systematic control due to the possibility of their self-loosening. In the case of the tested tank, this problem is negligible considering the low risk of cyclical loads. However, when determining their clamping force, it is required to take into account the microstructural heterogeneity of the steel plates. Applying too much force may result in catastrophic destruction of the puddled steel.
3. The presence of a considerable number of banded non-metallic inclusions in the material amid corrosive processes enables the formation of surface delamination and results in the appearance of characteristic corrosive “braids”. Such advanced corrosion means that installation of cathodic protection on the tank should be considered.
4. During operations, the tank has been subjected to uniform corrosion, which was favoured by the oxygen concentration cell (differential aeration). Corrosion pits were observed locally, reaching depths of approx. 1–1.5 mm. Under the thick “braids” of corrosive products on the sheet steel of the shell, the corrosion losses were approx. 1 mm deeper. The corrosion products of the sheets inside the tank did not contain sulphides with a reaction of pH = 6.5, so Microbiologically Influenced Corrosion caused by aerobic or anaerobic bacteria was not found in this case.
5. During renovation work, special attention should be paid to mechanical damage, including any physical impacts, as the material will be highly brittle due to the nature of the microstructure. Puddle joints also have much lower fatigue strength than modern steel joints. Therefore, numerical and non-destructive testing methods play an important role in their fatigue life prediction. Important recommendations in this regard are presented by Kuehn et al. [22].

The test results concerning the tank steel presented are a contribution to enriching the somewhat sparse test results related to properties of the steel used in 19th century building structures. This is relevant to heritage buildings which should be maintained in good technical condition, especially as some continue to be used. In particular, the presented results are helpful tools in the assessment and renovation of historic riveted structures.

## Figures and Tables

**Table 1:** Chemical composition of old steels in relation to results of own tests of tank steel—elemental content in [% mass].

Element	Puddled Steel Acc. to [12]	Cast Steel to 1906 Acc. to [12]	Current Requirements According to PN EN 10025-2:2019-11 [15]	Test Results for Tank Steel (Authors) Cont. [%] ± U
C	0.0032–0.15	0.026–0.20	<0.17	0.034 ± 0.054
Si	0.003–0.42	0.001–0.013	-	0.148 ± 0.044
Mn	0.054–0.18	0.036–0.52	-	0.120 ± 0.033
N	0.0037–0.04	0.011–0.022	-	-
P	0.011–0.39	0.0009–0.136	<0.035	0.115 ± 0.036
S	-	0.063–0.176	<0.035	0.010 ± 0.07

**Table 2:** Results of microanalysis of chemical composition performed in the area presented in Figure 15.

Element Symbol	Atomic Conc. [%]	Weight Conc. [%]	Uncertainty [%]
Pb	7.11	49.89	0.04
C	53.17	21.62	0.04
O	32.56	17.64	0.03
Fe	1.70	3.22	0.01
Zn	1.39	3.08	0.04
Ca	1.74	2.37	0.03
Si	1.81	1.72	0.03
Al	0.51	0.46	0.04

## Data Availability

The data presented in this study are available on request from the corresponding author.
